# Water in the upper mantle and deep crust of eastern China: concentration, distribution and implications

**DOI:** 10.1093/nsr/nwx016

**Published:** 2017-02-24

**Authors:** Qun-Ke Xia, Jia Liu, István Kovács, Yan-Tao Hao, Pei Li, Xiao-Zhi Yang, Huan Chen, Ying-Ming Sheng

**Affiliations:** 1 School of Earth Sciences, Zhejiang University, Hangzhou 310027, China; 2 CAS Key Laboratory of Crust-Mantle Materials and Environments, University of Science and Technology of China, Hefei 230026, China; 3 Hungarian Geological and Geophysical Institute, Budapest 1143, Hungary; 4 School of Earth Sciences and Engineering, Nanjing University, Nanjing 210023, China

**Keywords:** water, upper mantle and deep crust, continental stability, basalt genesis, eastern China

## Abstract

Understanding the concentration and distribution of water in the Earth's mantle plays a substantial role in studying its chemical, physical and dynamic processes. After a decade of research, a comprehensive dataset of water content in upper-mantle samples has been built for eastern China, which is now the only place with water-content data from such diverse types of natural samples, and provides an integrated picture of the water content and its distribution in the upper mantle at a continental scale. The main findings include the following: (i) the temporal heterogeneity of the water content in the lithospheric mantle from early Cretaceous (∼120 Ma) to Cenozoic (<40 Ma) was tightly connected with the stability of the North China Craton (from its destruction to its consolidation); (ii) the heterogeneous water content in the Cenozoic lithospheric mantle beneath different blocks of eastern China was not only inherited from tectonic settings from which they came, but was also affected later by geological processes they experienced; (iii) the distinct water content between the lowermost crust and lithospheric mantle of eastern China and its induced rheological contrast at the base of the crust indicate that the continental crust–mantle boundary could behave either in a coupled or decoupled manner beneath different areas and/or at different stages; (iv) the alkali basalts of eastern China demonstrate a heterogeneous distribution of water content in the mantle; local and regional comparisons of the water content between the lithospheric mantle and basalts' source indicate that the Cenozoic alkali basalts in eastern China were not sourced from the lithospheric mantle. Instead, the inferred high water contents in the mantle sources suggest that the Cenozoic eastern China basalts were likely sourced from the mantle transition zone (MTZ); and (v) both oceanic and continental crusts may carry a certain amount of water back into the deep mantle of eastern China by plate subduction. Such recycled crustal materials have not only created a local water-rich zone, but have also introduced crustal geochemical signatures into the mantle, both accounting for crustal geochemical imprints in the intra-plate magmatic rocks of eastern China.

## INTRODUCTION

Hydrogen that is structurally bound to other ions (mainly oxygen) in minerals is traditionally referred to as ‘water' in the Earth science community and its concentration is calculated as H_2_O by weight. Its existence, even at trace levels (ppm level), can significantly influence certain important chemical and physical properties (e.g. seismic velocities, electrical and thermal conductivities, rheology, optical properties, melting temperature, pressure and phase relations and ion diffusion rate) of minerals and therefore rocks [1–13]. Consequently, water affects the chemical, physical and dynamic processes of rocks and their domains in the deep Earth, such as the relative movement of plates and the genesis and evolution of magmas [14–24]. Moreover, the amount of water in the continental lithospheric mantle is predicted to be closely related to its viscosity and stability [25,26]. Investigations on natural samples have demonstrated that an elevated water content can induce the destruction of cratons [27], whereas a reduced water content ensures craton longevity [22,28]. Therefore, understanding the concentration and distribution of water in the mantle plays a substantial role in explaining its formation and evolution. Furthermore, knowledge about the water content in the source of mantle-derived magmas may provide new constraints on the geochemical heterogeneities of the mantle and the involved processes [29,30].

**Figure 1.**
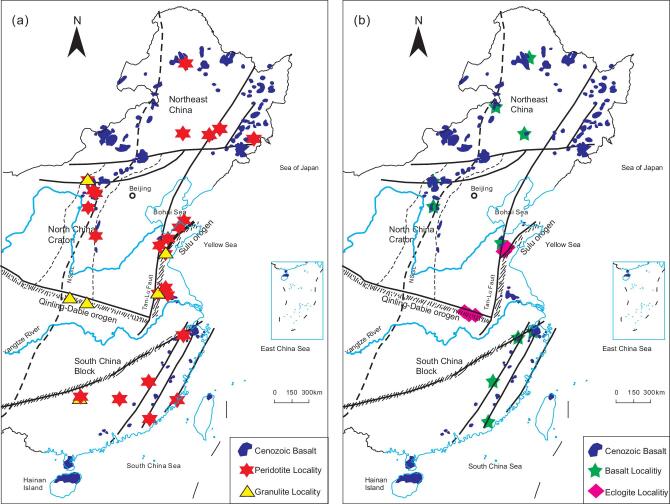
Sample locations in eastern China. (a) Peridotite and granulite locations; (b) basalt and eclogite locations.

The concentration and distribution of water in the deep lithosphere (lower crust and lithospheric mantle) and asthenospheric mantle can be determined by natural samples, including mafic granulites, peridotites, pyroxenites, eclogites and basalts, although information about the mantle transition zone (MTZ) and lower mantle is mostly constrained by high-temperature and -pressure experiments [31]. Recently, after a decade of research, a comprehensive dataset of the water content in peridotite and granulite xenoliths hosted by alkali basalts, terrain granulites, ultrahigh pressure metamorphic (UHPM) eclogites and Mesozoic-Cenozoic alkali basalts has been built for eastern China, which is now the only place with water-content data from such diverse types of natural samples (Fig. 1). This dataset provides an integrated picture of the water content and its distribution in the lower crust and upper mantle at a continental scale. In this review, we do not intend to compare our dataset from eastern China to those of other localities in the world because such comparisons have been covered well by Peslier [32] and Demouchy and Bolfan [33]. Instead, we will focus on the temporal and spatial distribution of water in the lower crust and upper mantle beneath eastern China and will summarize the origins of their heterogeneities and implications. Our primary purpose is to provide an example to address the water distribution and its role in the deep Earth at a continental scale.

## ANALYTICAL METHODS OF WATER CONTENT

The main minerals in the Earth's upper mantle and lowermost crust include olivine (ol), clinopyroxene (cpx), orthopyroxene (opx), garnet (grt) and plagioclase (pl), and their high-pressure polymorphous. They are nominally anhydrous minerals (NAMs), meaning that there is no hydrogen in their stoichiometric chemical formulas. However, hydrogen can be incorporated into the structure of NAMs, usually as charge-compensating cation(s) often coupled with other cations (such as Al^3+^ or Ti^4+^). Hydrogen in such vacancies is normally bonded to one of the coordinating oxygens and forms a hydroxyl group [34], of which the content is expressed as H_2_O by weight ppm (hereafter referred to as ppm). Although the water content of minerals in the upper mantle and lowermost crust is generally less than several hundreds of ppm, they are likely the main water reservoir due to their predominant mass and volume proportions [32].

Fourier Transform Infrared Spectroscopy (FTIR) and Secondary Ion Mass Spectrometry (SIMS) are the most commonly used methods to measure the water content in NAMs [35]. Until now, all of the published water contents in mineral constituents of peridotites, granulites and eclogites from eastern China have been obtained using FTIR, with the exception of Aubaud *et al*. [36], who analysed several ol and pyroxene grains in Hannuoba peridotite xenoliths hosted in the Cenozoic basalts with SIMS. The SIMS data from Aubaud *et al*. [36] are comparable to the FTIR data of Yang *et al*. [37] from the same location. The water contents of the whole rocks, if reported, were estimated from the mineral water contents and their respective modal proportion through mass balance calculations. The water contents of basaltic magmas were calculated from the water content of cpx phenocrysts and the partition coefficient of water between cpx and basaltic melt. The details of the applied FTIR methodology can be found in Rossman [35], Kovács *et al*. [38], Xia *et al*. [27] and Demouchy and Bolfan [33]. The detailed run conditions and procedures have been described in the cited references. In short, the uncertainty of the measured water content of minerals in peridotites, granulites and eclogites was generally less than 20%, and that of the water content of basalts calculated from the water content of cpx phenocrysts was generally less than 40%. The factors influencing the uncertainty and the evaluation methods can be found in the cited references.

## WATER IN THE LITHOSPHERIC MANTLE OF EASTERN CHINA

### FTIR spectra and water contents

The eastern Chinese continent consists of three main blocks from north to south: the Northeast China (NEC), the North China Craton (NCC) and the South China Block (SCB). Numerous small-volume basaltic volcanoes are distributed in these blocks and many contain abundant peridotite xenoliths, providing a good opportunity to investigate water distribution in the lithospheric mantle of eastern China. The water contents in the main constitute minerals (cpx, opx, ol and grt) in peridotite xenoliths from 28 basaltic localities, extending from Heilongjiang province in the north to Hainan province in the south (Fig. 1), have already been reported [37, 39–47]. Most of the studied peridotite xenoliths are spinel facies peridotites, except a few samples from the Nuomin volcano in the NEC and the Mingxi volcano in SCB, which are garnet facies peridotites [42,45].

FTIR investigations have shown that the garnets and most of the ol usually display no OH-related absorption bands, but there are also ol from several localities (Mingxi, Anyuan, Niutoushan and Qilin) in SCB that have prominent OH bands at 3575 cm^−1^, 3520 cm^−1^, 3340 cm^−1^ and 3230 cm^−1^. In contrast, except for the Nuomin peridotites, all of the opx and cpx show several OH absorption bands: 3595–3570 cm^−1^, 3525–3500 cm^−1^, 3415–3390 cm^−1^ and 3315–3300 cm^−1^ for opx, and 3635–3600 cm^−1^, 3550–3510 cm^−1^ and 3470–3445 cm^−1^ for cpx. The positions of these bands for ol, cpx and opx have been attributed to the vibration of the structural OH and are similar to those reported in peridotites worldwide [34,48–56]. It is particularly interesting that all of the ol, grt, cpx and opx grains in 13 peridotites from the Nuomin volcano in NEC have no any detectable OH band, demonstrating a very dry lithospheric mantle [45].

The water contents of minerals in the peridotites range from approximately 0 to 41 ppm for ol, 0 to 346 ppm for opx and 0 to 746 ppm for cpx. Except for some opx grains from the Panshishan and Tianchang basalts in the NCC [45,57], cpx and opx in peridotite xenoliths from other localities exhibit homogeneous water contents within individual grains, regardless of grain size. Moreover, the water content in opx usually displays positive correlations with the Al content [45,58]. Therefore, it is very likely that the cpx and opx retain the original water content that is typical for their mantle source. However, diffusional loss of hydrogen during peridotite ascent to the surface in the host basaltic magmas cannot be excluded for ol, considering near zero water in most of the ol grains. If D_cpx/ol_ = 10 is assumed, based on experimental results [20,21,36,39,59–63], the estimated water contents of the whole rocks using the calculated ol water content and the respective modal proportions of minerals range from 0 to 225 ppm, which cover the range of the Mid-ocean ridge basalt (MORB) source (50–200 ppm) [19,64,69] and are less than the ocean island basalt (OIB) source (300–1000 ppm) [68,70–74]. The water contents of the peridotites in eastern China vary among samples, even within a single locality. For example, the water contents range from 5 to 140 ppm, 5 to 355 ppm and 2 to 72 ppm in opx, cpx and whole rock, respectively, in peridotites from the Nushan volcano [37]. Furthermore, the water contents of the peridotites vary among different localities. For instance, the Nuomin peridotites in the NEC have no detectable water; the Hannuoba peridotites in the NCC have relatively low water content, ranging from 20 to 55 ppm, 50 to 150 ppm and 10 to 35 ppm for opx, cpx and whole rock, respectively; and the Jiande peridotites in the SCB contain much more water with 163 to 329 ppm, 388 to 589 ppm and 85 to 216 ppm for opx, cpx and whole rock, respectively [37,44].

**Figure 2.**
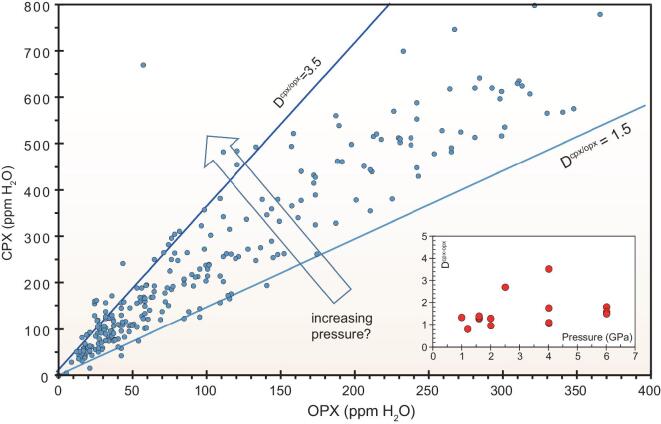
Cpx water contents versus opx water contents in peridotite xenoliths of eastern China. The data are from Aubaud *et al*. [36], Yang *et al*. [58], Hao *et al*. [43–45,78], Li *et al*. [47], Wang *et al*. [46], Xia *et al*. [40,41] and Yu *et al*. [42]. The inserted plot shows the correlation between D_cpx/opx_ with pressure in experimental studies [21,36,62,76,77].

Most of the ol grains in the peridotite xenoliths from eastern China have no detectable water; this is likely a result of the diffusional loss of hydrogen from ol during ascent, so the partition coefficient of water between ol and other minerals cannot be evaluated. The correlation of water content between cpx and opx is shown in Fig. 2 [36,40–47,58]. The ratios of H_2_O between cpx and opx fall in a range from 1.5 to 3.5 and can be regarded as partition coefficients (D_cpx/opx_), since cpx and opx have preserved the original water content that is typical for their mantle source. The D_cpx/opx_ of the peridotites in eastern China has a similar range to those from natural peridotite xenoliths worldwide [22,34,50,52,53,55,56,75]. From the available experimental results [21,36,62,76,77], it appears that D_cpx/opx_ increases with increasing pressure (see the inserted plot in Fig. 2), so the variation in D_cpx/opx_ in peridotites worldwide may be a proxy for their depth of origin within the lithospheric mantle. If this is correct, it supports the common view of mantle petrologists and geochemists that the occasionally captured peridotite xenoliths by alkali magmas can well represent the entire lithospheric mantle, which is often questioned by geophysicists and geologists.

### Regional heterogeneity in the Cenozoic lithospheric mantle

**Figure 3.**
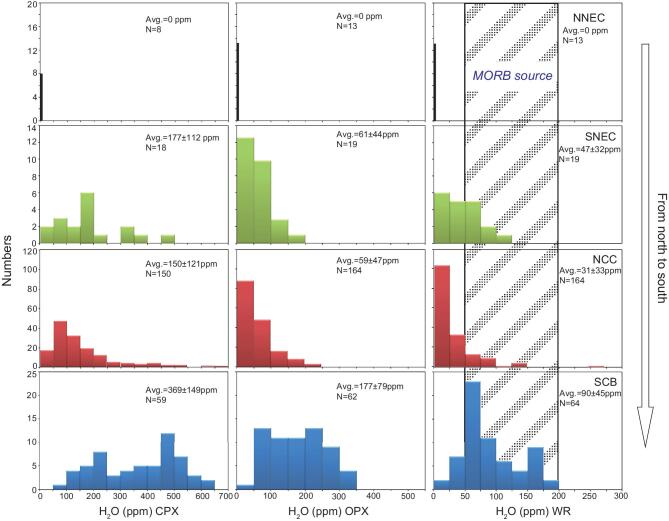
Comparison of the water contents of cpx, opx and whole rock of the peridotite xenoliths in different blocks from eastern China. The data sources are the same as in Fig. 2.

The regional distribution of the water content in the Cenozoic lithospheric mantle beneath all of eastern China, from the north part of the NEC (NNEC) to the SCB, has been discussed by Hao *et al*. [45] and is shown in Fig. 3. The Nuomin peridotite xenoliths are absolutely dry, indicating a dry lithospheric mantle beneath the NNEC. The lithospheric mantle of the south part of the NEC (SNEC) and the NCC is characterized by lower water contents than the MORB source, and the SNEC and the NCC share similar ranges and average values. The lithospheric mantle of the SCB has much higher water content than other regions in eastern China, falling in the range of the MORB source. By integrating the water content with other geochemical indices, Hao *et al*. [45] suggested that the regional variations in the water content of the lithospheric mantle beneath eastern China cannot be caused by distinct partial melting and mantle metasomatism events or the redox state. Instead, the lithospheric mantle beneath different regions may have distinct origins and have undergone distinct geodynamic processes. The NNEC lithospheric mantle is supposed to be from the Siberia craton, with hydrogen diffused out of the peridotite minerals when the Siberia craton had interacted with a super mantle plume. The relatively low water content of the NCC and the SNEC may have been caused by the reheating effect of an upwelling asthenospheric flow during the lithospheric thinning event in the Mesozoic. The higher water content of the SCB peridotites suggests that the large part of its deeper lithospheric mantle was accreted from the asthenosphere, and either the SCB did not undergo a significant lithospheric thinning event such as the NCC or the thinning mechanism was different. From the case of eastern China, it seems that water distribution in the lithospheric mantle may provide new constraints on the origins and geodynamic processes of continents.

### Temporal heterogeneity in the NCC lithospheric mantle

Although the spatial distribution of water content and the controlling factors in the lithospheric mantle are of increasing interest to the Earth science community, less attention has been paid to the temporal variation of water content, which also matters greatly and may provide new insights into the evolution of the lithospheric mantle. Abundant mantle xenoliths entrained in the alkali basalts with a wide range of eruption ages (125 to < 40 Ma) across the NCC, especially the eastern part, provide a good chance to address that issue.

A detailed investigation of the bulk water content of peridotite xenoliths from many localities indicates that the Cenozoic lithospheric mantle (<40 Ma) beneath the eastern part of the NCC was relatively dry, with an average of 25 ± 18 ppm (from 15 to 85 ppm), except for a few localities close to the deep-cutting Tan-Lu fault [40,45,58]. The extremely low bulk water content below 50 ppm compared to the samples from other continental lithospheric mantles worldwide that are typical for cratonic and off-cratonic peridotites (normally 40–180 ppm, with average values of 119 ± 54 ppm and 78 ± 45 ppm, respectively) and sub-oceanic mantle lithosphere values (>50 ppm) inferred from MORB and OIB (see the compiled dataset in Xia *et al*. [40]) highlights the uniqueness of the eastern part of the NCC and implies its links with the destruction of the NCC, as stated in the previous section.

Li *et al*. [47] reported the water content of the peridotite xenoliths hosted by the late Mesozoic basalts (82–67 Ma) from eastern NCC, revealing two types of mantle domains with contrasting bulk water contents. The lithospheric mantle domain, represented by the Daxizhuang xenoliths at 74 Ma, was relatively ‘dry', sharing similar characteristic with the Cenozoic lithospheric mantle [40,47,58]. Meanwhile, the peridotite xenoliths from Junan (67 Ma) and Qingdao (82 Ma) suggest a relatively high bulk water content for the lithospheric mantle, with an average of 130 ± 20 ppm, which is in the range of the MORB source (50–250 ppm). When mantle xenoliths are scarce, the bulk water content of the lithospheric mantle can also be obtained indirectly. Based on a study of water in the early crystallized cpx phenocrysts in basalts, Xia *et al*. [27] estimated that the bulk water content of the lithospheric mantle source of the Feixian high-magnesium basalts. The bulk water content was more than 1000 ppm, which is several times higher than that of the MORB source, demonstrating that the NCC lithospheric mantle was very water-enriched in the early Cretaceous (∼120 Ma), at the paroxysm of the destruction of the NCC.

**Figure 4.**
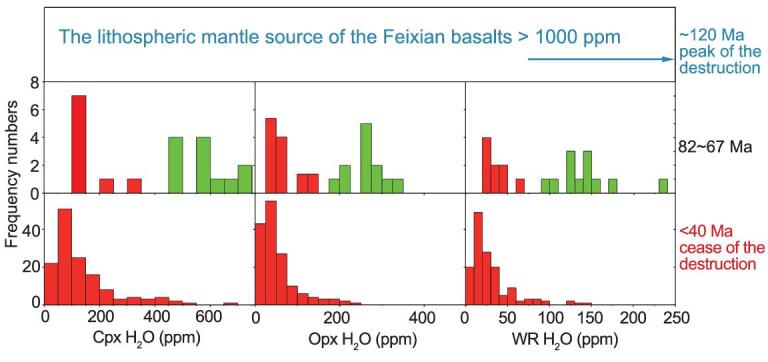
The temporal variation of the bulk water content in the lithospheric mantle beneath the eastern NCC. The figure is modified from Fig. 11 in Li *et al*. [47] by adding the cpx water contents and the data sources are the same as Li *et al*. [47].

These combined snapshots envisage a temporal variation of water content for the eastern NCC lithospheric mantle (Fig. 4) [47]. At the peak of the destruction (∼120 Ma), the lithospheric mantle was hydrous; after its destruction was complete at the end of the Mesozoic, the water content of the lithospheric mantle was extremely low; during the period of destruction, the lithospheric mantle had intermediate water content [47]. Li *et al*. [47] further discussed the process of this cratonic destruction as follows: (i) at the peak time of destruction, the hydrated, water-rich lithospheric mantle offers a presumably low-viscosity state for its own destruction; (ii) during the destruction, the upwelling asthenosphere erodes the lowermost lithospheric mantle and continuously heats the overlying lithospheric mantle, which results in lithospheric thinning and dehydration of the relict lithospheric mantle. This goes on until the cessation of the destruction of the cratonic lithospheric mantle, which is, at least in part, due to its now lower water content and stiffer rheology, therefore supporting better resistance to further tectonic removal. The upwelling asthenosphere, however, cools and is transformed into a newly accreted lithospheric mantle with probably higher bulk water content than that of the dehydrated relict cratonic lithospheric mantle.

These findings above seem to contrast with those of Liu and Xia [79], who reported the water content of six peridotite xenoliths entrained in the early Cretaceous (∼125 Ma) high-Mg diorite from Fushan in the western part of the NCC, which show much lower water content than their hydrated, more water-rich Mesozoic counterparts in the eastern part of the NCC. The water content in cpx and opx ranges from 216 to 404 ppm and 123 to 188 ppm, respectively, with an average of ∼40 ppm for whole rock, which is far less than >1000 ppm of the Feixian mantle source but is in the range of the classic craton [34,50,80,81]. The contrasting water contents could be interpreted as the hydration of the NCC lithospheric mantle by the westward subduction of the paleo-Pacific plate, which may not have yet reached the western part of the NCC by the early Cretaceous.

## WATER IN THE LOWER CONTINENTAL CRUST OF EASTERN CHINA

### Temporal variations in water content of the lower continental crust

The lower continental crust, separating the shallow crust from the underlying lithospheric mantle, is of crucial importance in determining the tectonic evolution of continents and in buffering the exchange of materials between Earth's interior and exterior. Despite great efforts of water in mantle minerals, much less attention has been paid to the speciation and concentration levels of water in the lower crust and its bearing on physical properties.

**Figure 5.**
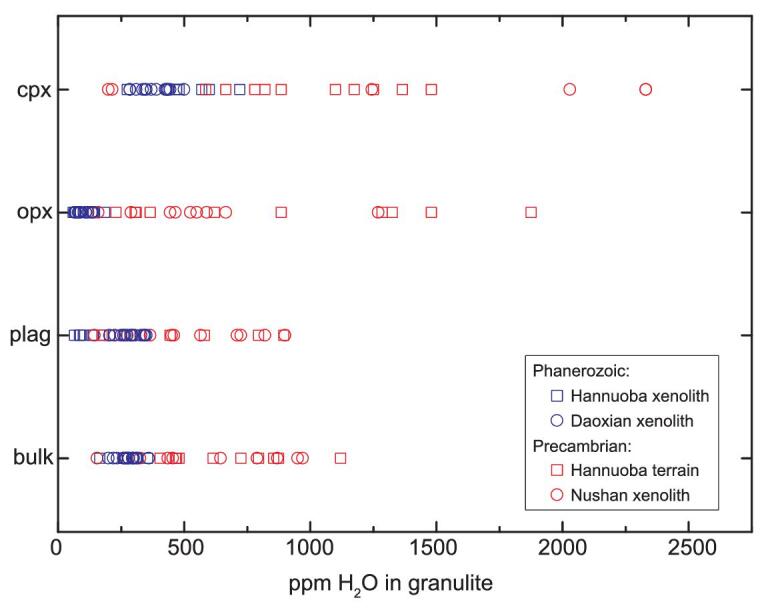
Water contents of the lower crustal minerals in eastern China and their temporal contrast (after Yang *et al*. [37]).

A survey by FTIR on xenolith and terrain granulites, typical of samples from the lower crust, from Hannuoba, Nushan and Daoxian, in eastern China, has shown that the main constitutive minerals, such as plagioclase (pl), cpx, opx and grt, commonly contain trace amounts of OH in their lattice structure, also including molecular H_2_O for some pl [58,82]. Thus, minerals in the lower crust have a similar ability to dissolve water as minerals in the upper mantle, although its concentration may be quite different (as shown below). The calculated water concentrations of lower crustal granulites range from approximately 200 to 2000 ppm for cpx, 60 to 1800 ppm for opx, 65 to 900 ppm for pl, 300 to 600 ppm for grt and 150 to 1100 ppm for the bulk rocks (Fig. 5) [37]. Recently, Németh *et al*. [83] measured the water contents of minerals in a suite of granulite xenoliths from the Pannonian Basin in east-central Europe; their data demonstrate the occurrence of approximately 0–271 ppm, 55–413 ppm and 0–684 ppm for cpx, opx and pl, respectively. These values are much smaller than those of other granulites from eastern China. Therefore, it appears that the distribution of water is highly heterogeneous in the lower crust, on both small (i.e. different samples from the same locality) and large (i.e. different localities) scales. This is similar to the observed chemical heterogeneities of many elements and isotopes in samples from the lower crust (e.g. Rudnick and Gao [84] and references therein). Such heterogeneities are presumably either inherited from the protoliths or they reflect the complicated history of crust–mantle interactions.

An interesting result arising from the available data is that, by classifying the samples according to their formation ages, pre-Phanerozoic granulites record apparently higher water contents than Phanerozoic granulites (Fig. 5) [37]. One possible cause is that the early lower crust was more hydrous than the modern one, although it is not clear whether this is a local phenomenon or a general trend on a global scale [37]. For the granulite samples compiled in Fig. 5, the chemical composition is broadly similar for individual minerals, and so are the temperature and pressure conditions of equilibrium [37]. Therefore, a significant contrast in water content in these samples deserves some further discussion, even if Yang *et al*. [37] already provided some clues from a petrological perspective. Recently, Yang [85] experimentally demonstrated how, at a given pressure and temperature, the ability of pl in incorporating OH is more significant under very reduced conditions, e.g. at four or more log units below the QFM (quartz-fayalite-magnetite) buffer. Considering the thermodynamics of OH dissolution in silicate minerals [86] and the redox state of around QFM in the modern lower crust [87], the observed water contrast in Fig. 5 can be explained by a change of the prevailing redox state in the lower crust, in that the pre-Phanerozoic lower crust of eastern China was regionally more reduced. Alternatively, it is possible, considering the even lower water contents of granulite xenoliths from the younger Miocene Pannonian Basin in Central Europe [83], that the observed lower water content in the Phanerozoic Chinese xenolith may be related to repeated depletion events since the Precambrian. This appears to be a logical explanation, as the continental crust may have remained stable over geologic time, whereas the continental lithospheric mantle may have been removed during cratonic destruction. This means that the water budget in the continental lower crust may have decreased gradually over geologic time during repeated melt removals and heating episodes during extension and that it could only be rehydrated locally, as suggested by Németh *et al*. [83].

### Vertical distribution of water content in the lower crust and lithospheric mantle

The vertical distribution of water content in the shallow mantle has received increasing attention in recent years, and has been addressed in some studies [28]. However, the variation in water content from the lithospheric mantle to the overlying lower crust has not been well documented, even if it may be of equal importance for the stability and tectonics of the continents.

**Figure 6.**
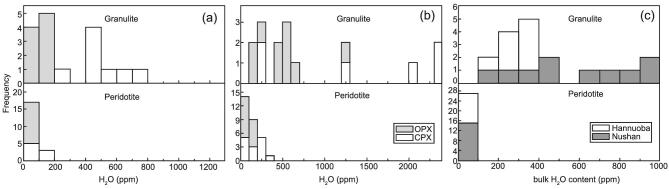
Vertical distribution of water content in the lower crustal granulite xenoliths and mantle peridotite xenoliths. (a) The cpx and opx in the Hannuoba xenoliths; (b) the cpx and opx in the Nushan xenoliths; and (c) the bulk water content (after Yang *et al*. [58]).

Yang *et al*. [58] addressed this question with reported data of water contents from both granulite xenoliths and peridotite xenoliths from Hannuoba and Nushan within the NCC (eastern China). They found much higher water contents in both the mineral constituents and bulk rocks in granulites than in peridotites (Fig. 6) [58]. The contrast of water content may be related to petrological processes and histories of these rocks, such as partial melting, differentiation and metamorphism; however, thermodynamically, it may indicate that the incorporation mechanism of OH in lower crustal minerals is different from the mechanism in mantle minerals, e.g. due to different pressure, temperature and mineral chemistry (lower crustal pyroxenes are richer in Fe and Al than their mantle counterparts) [58].

The vertical variation in water content in the lower crust and sub-continental lithospheric mantle and their lateral differences, as shown in Fig. 6, allows us to explore the rheological properties of the deep lithosphere in eastern China. Considering the data on water and regional thermal (*P*–*T*) conditions, it appears that the lower crust is much stronger than the underlying shallow lithospheric mantle at Hannuoba but weaker at Nushan. This makes the lithosphere thermally and mechanically unstable at Hannuoba compared to Nushan. This indicates that, during the Cenozoic when the xenoliths were brought to the surface, the lithosphere may have undergone mechanical thinning at Hannuoba but thickening at Nushan [58]. This means that different lithospheric processes may have been operative in different tectonic areas beneath eastern China and that the variation in water content in the deep lithosphere is critical for understanding deep processes.

## WATER IN THE CENOZOIC ALKALI BASALTS OF EASTERN CHINA

In eastern China, from Wudalianchi in Heilongjiang Province in north-eastern China to the Hainan Island in the south end of mainland China, the Cenozoic basalts are widely distributed. These basalts generally exhibit typical OIB-like trace element patterns and Sr–Nd isotopic compositions. The enriched components in the mantle sources of the Cenozoic basalts in eastern China are still not fully understood. The old lithospheric mantle, the recycled ancient lower continental crust and recycled oceanic crust in the asthenosphere were all suggested to be responsible for their formation [88–93]. Recently, the materials in the hydrated MTZ beneath eastern Asia were also proposed to be involved in the mantle sources of the basalts in north-eastern China [94,95]. It has been widely accepted that the water content of magma, and the corresponding H_2_O/Ce ratios, would be useful for identifying the mantle components [96–98]. The importance of water in our understanding of the genesis of alkali basalts in eastern China was also invoked as early as 2007 [99]. However, there has been minimal research trying to analyse the water contents of these basalts, except several attempts to argue that basalts from several locations may be rich in water that used thermodynamic calculations [95,100] or an indirect inference from the low δ^18^O values of mineral phenocrysts [101]. Only very recently were the water contents of the Cenozoic alkali basalts, from north-eastern to south-eastern China, constrained [29,30,102–105]. These results are based on a method that relies on the water contents of cpx phenocrysts and chemical composition-dependent water partitioning between cpx and basaltic melts, which was first suggested by Wade *et al*. [106] and considerably improved by Xia *et al*. [27] and Liu *et al*. [30]. Here, we summarize the distribution of the water contents in these alkali basalts in eastern China determined by this method and its inferences about the sources of the basalts, enriched components in the sources and implications about the role of the subducted Pacific slab on the genesis of the Cenozoic basalts in eastern Asia.

### Regional distribution of H_2_O and H_2_O/Ce

**Figure 7.**
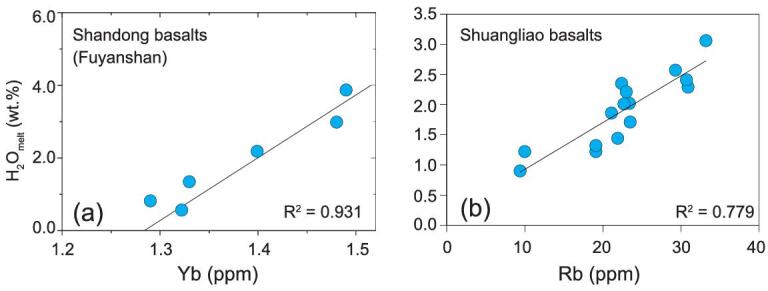
The correlation between the calculated magma water contents and the bulk rock trace element concentrations. The data for the Shandong and Shuangliao basalts are from Liu *et al*. [104] and Chen *et al*. [29], respectively.

It is worth mentioning that the uncertainty of this methodology may be up to ∼40% [30,41]. To recover the water content of the Cenozoic alkali basalts, cpx phenocrysts with Mg# (=100* Mg/(Mg+Fe), mol percent) >75 were used [29,30,104]. Although multiple processes, such as crystallization, crustal contamination, degassing and diffusion of hydrogen out of cpx during or after eruption, might affect the final calculated magma-water contents, reasonable correlations between the calculated water contents and incompatible element contents of bulk rocks (Fig. 7 [29,103] shows examples from the Shandong and Shuangliao basalts) indicate that none of these processes may have a significant impact [29,104]. The H_2_O/Ce ratios were determined by the calculated water contents and the measured Ce concentration of the bulk rocks, which are rather consistent with the average H_2_O/Ce values calculated by $\frac{{C_w^{cpx}/D_w^{cpx/melt}}}{{C_{Ce}^{cpx}/D_{Ce}^{cpx/melt}}}$ for a group of cpx phenocrysts, where C is the concentration of H_2_O and Ce, and D is the partition coefficient between cpx and basaltic melt [104].

**Figure 8.**
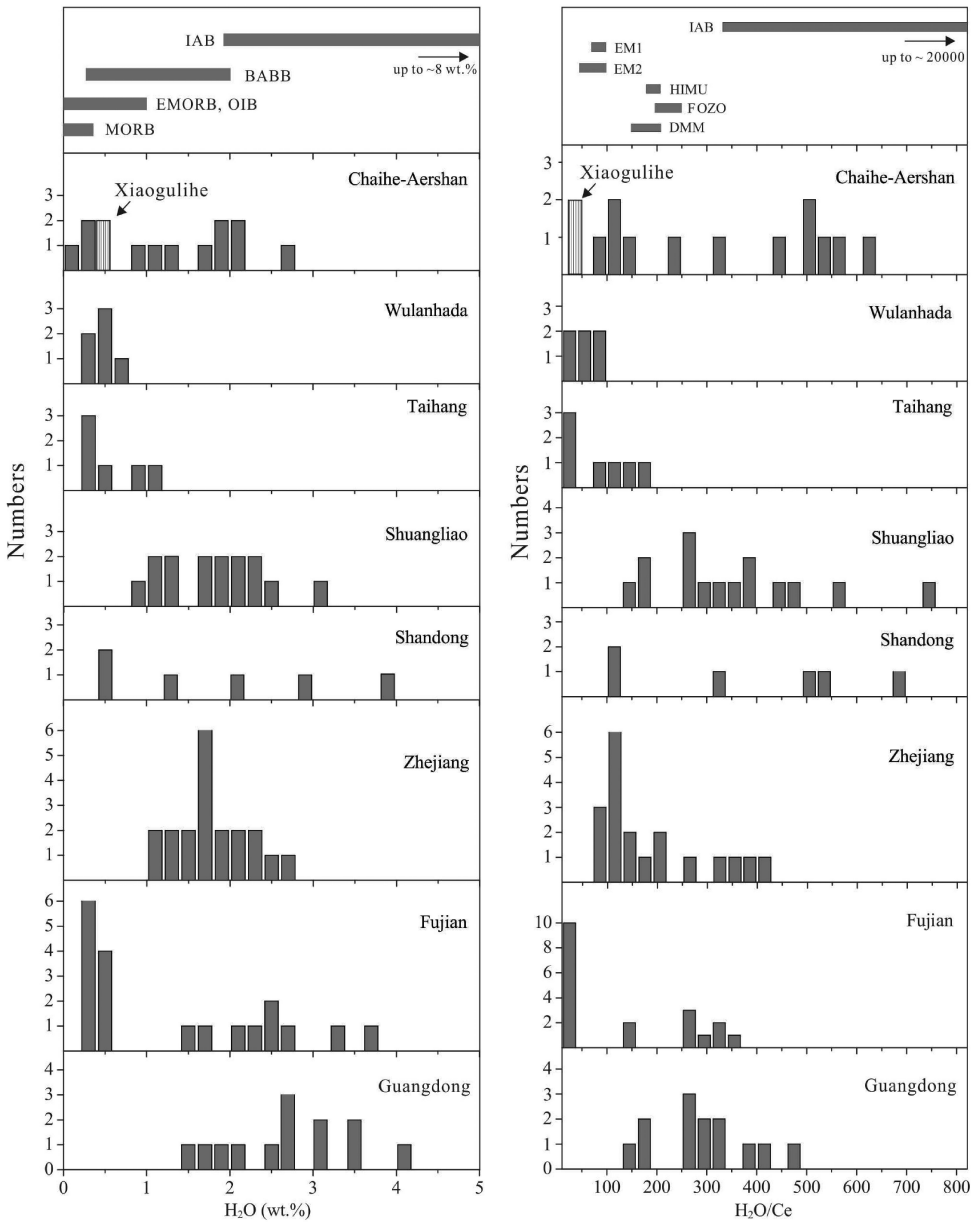
The water contents and H_2_O/Ce ratios of the Cenozoic alkali basalts in eastern China (after Chen *et al*. [121]). The data are presented from northern to southern localities from top to bottom. The water content data of the Cenozoic basalts in eastern China are from Chen *et al*. [29,102,103,121], Liu *et al*. [30,104] and Liu *et al*. [105]. The data source for IAB, BABB, OIB, MORB and E-MORB is from Asimow *et al*. [64], Danyushevsky *et al*. [107,108], Dixon and Clague [97], Dixon *et al*. [65,70,98], Dobson *et al*. [109], Hochstaedter *et al*. [110], Michael [66,96], Nichols *et al*. [71], Saal *et al*. [67], Simons *et al*. [68], Sisson and Layne [110], Sobolev and Chaussidon [69], Stolper and Newman [112] and Wallace [113,114]. The H_2_O/Ce ratio of PM is calculated with H_2_O from Dixon and Clague [97] and Ce from Sun and McDonough [115], and the H_2_O/Ce ratio of the EM1, EM2, HIMU, FOZO, DMM and IAB is from Cabral *et al*. [116], Dixon *et al*. [98], Kendrick *et al*. [117,118], Workman *et al*. [74] and Ruscitto *et al*. [119].

**Figure 9.**
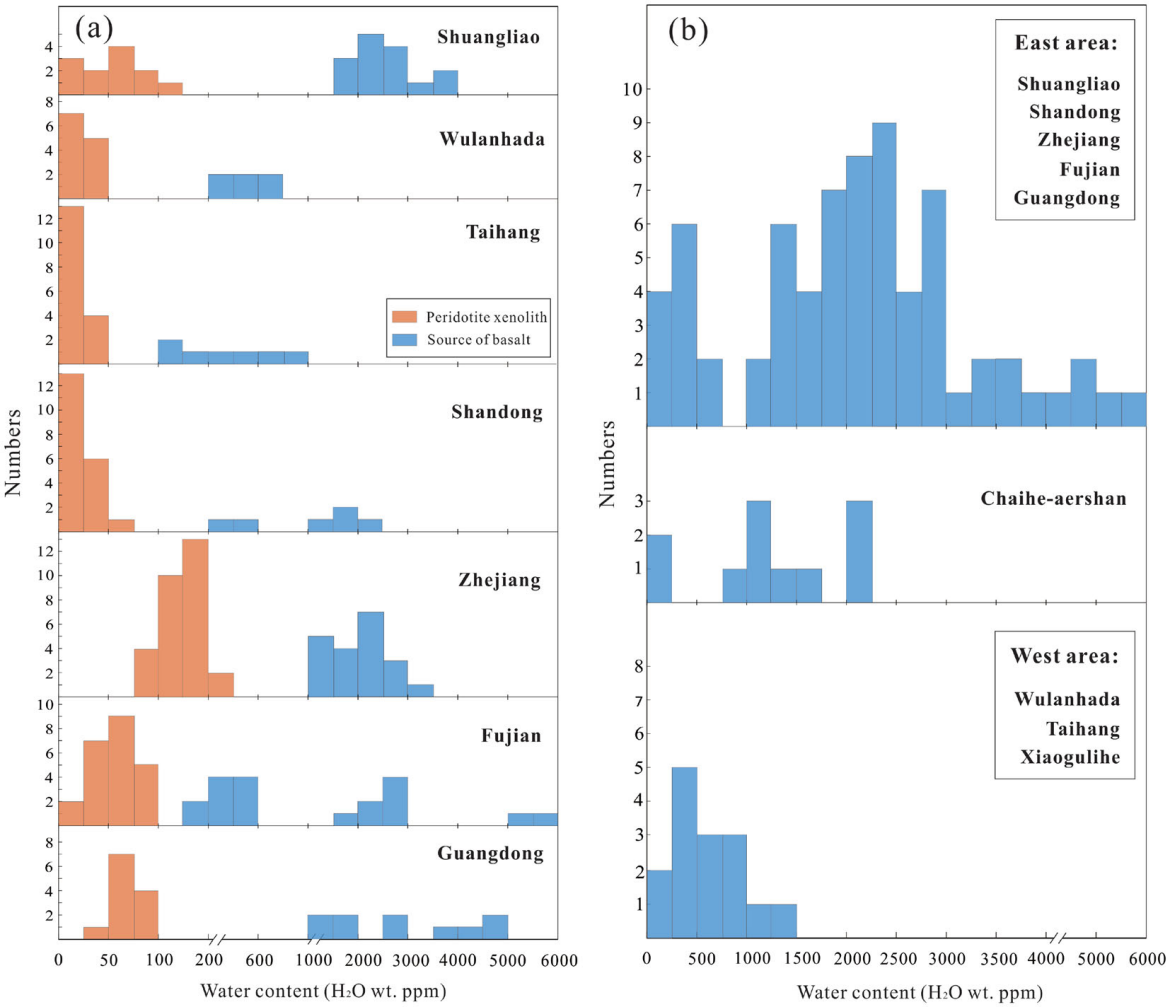
(a) Comparison of the water contents in the sources of the Cenozoic eastern China basalts with those in the peridotite xenoliths from the same regions. (b) The spatial distribution of the water content in the sources of the Cenozoic eastern China basalts. After Chen *et al*. [121].

The water contents and H_2_O/Ce ratios of the alkali basalts from different locations in eastern China are statistically shown in Fig. 8 [29,30,64–68,70,71,74,96–98,102–104,107–119], from which local and regional scale heterogeneities can be identified. The recovered magma-water contents varied from 0.6 to 3.9 wt.% in the Shandong basalts, from 0.9 to 3.1 wt.% in the Shuangliao basalts, from 1.1 to 2.7 wt.% in the Zhejiang basalts, from 0.2 to 3.8 wt.% in the Fujian basalts and from 1.6 to 4.2 wt.% in the Guangdong basalts. The higher water contents fall in the range of back-arc basin basalts (BABB) and island arc basalts (IAB), even considering the ∼40% uncertainty. By contrast, the water contents of the basalts in Taihang and Wulanhada in Central NCC and Xiaogulihe in the northern part of NE China are much lower (0.2–1.4 wt.%, 0.21–0.69 wt.% and ∼0.5 wt.%, respectively), which are close to the water contents of MORB and typical OIB. Note, the water contents found in the Wulanhada basalts do not support the conclusion that the basalts in this area are very hydrous [100,120], which was deduced from non-analytical approaches (the MELTs program modeling or indirect evidence from the involvement of hydrated lower oceanic crust, respectively). When the results from direct measurements are considered, it seems that the basalts in the region closer to the Pacific trench (Shuangliao, Shandong, Zhejiang, Fujian and Guangdong, referred to as the eastern area in Fig. 9) [120] exhibit higher water content in the magma than those further away from the trench (Xiaogulihe, Wulanhada and Taihang, referred to as the western area in Fig. 9). However, as an outlier, the water contents of the Chaihe-Aershan basalts in NE China span a similar range with those of the Shandong and Shuangliao basalts, although they have comparable distance to the Pacific trench as to the Wulanhada and Xiaogulihe basalts. Correspondingly, the H_2_O/Ce ratios of basalts exhibit a similar regional distribution (Fig. 8). When the maximum and mean values of the H_2_O/Ce ratios of the Shandong, Shuangliao, Zhejiang, Fujian, Guangdong and Chaihe-Aershan basalts are obviously higher than those of the most water-rich OIB (∼250) [98], those of the Taihang, Wulanhada and Xiaogulihe basalts are close to or significantly lower than that of the normal MORB (∼200–210) [122].

### Where did water come from?

Whether the Cenozoic basalts in eastern China are from the lithospheric mantle is still under fierce debate after more than three decades of intensive study [30,89,91,92,104,123–127]. Systematic investigations of the water contents of the alkali basalts and their hosted peridotite xenoliths that represent the lithospheric mantle may provide new insights. More precisely, our dataset on the water contents of eastern Chinese samples allow us to make both local and regional comparisons. This means that the water-content contrast between the basalt source and the lithospheric mantle for a specific area, and the regional water distribution in the lithospheric mantle and in the basalt source for entire eastern China could be explored.

Chen *et al*. [121] have estimated the water contents in the sources of the Cenozoic eastern China basalts using the following: (i) the calculated water contents of the initial basaltic melts; (ii) the available partition coefficients of water between basalts and mantle rocks; and (iii) the estimated partial melting degrees that the basalt source experienced. The mantle source-water contents ranged between approximately 150 and 4700 ppm and most of them were higher than 500 ppm (Fig. 9b), regardless of the used partial melting model (batch melting or fractional melting) [121]. By comparing the estimated water contents in the basalt sources with those in the peridotite xenoliths [121], the flowing can be clearly determined: (i) the source-water contents for all the basalts are significantly higher (several to several hundred times) than those of the lithospheric mantle in the same region (Fig. 9a); (ii) except for the Chaihe-Arershan basalts in the north-west NEC, the water contents of the basalt sources appear to decrease from the east to the west (Fig. 9b), which is different from the water-content trend in the lithospheric mantle of eastern China decreasing from the south to the north (Fig. 3). Thus, the local and regional differences in the water contents in the lithospheric mantle and the basalt sources indicate that the Cenozoic basalts in eastern China may not have been derived from the lithospheric mantle.

The upper mantle (except the lithospheric mantle containing amphibole or mica) can only accommodate 50–250 ppm of H_2_O [19,31,128], and the lower mantle contains less or similar amounts of water [128,129]. Only the MTZ can contain several hundred ppm to >10 000 ppm H_2_O [128,130]. Therefore, the estimated water contents (150–4700 ppm, and most of them higher than 500 ppm) in the sources of the Cenozoic eastern China basalts are likely from the MTZ. This proposition is compatible with geophysical investigations that have shown the stagnated oceanic slabs in the MTZ beneath eastern Asia [131,132], which are expected to provide water once they are disturbed.

### Enriched components in the mantle source

**Figure 10.**
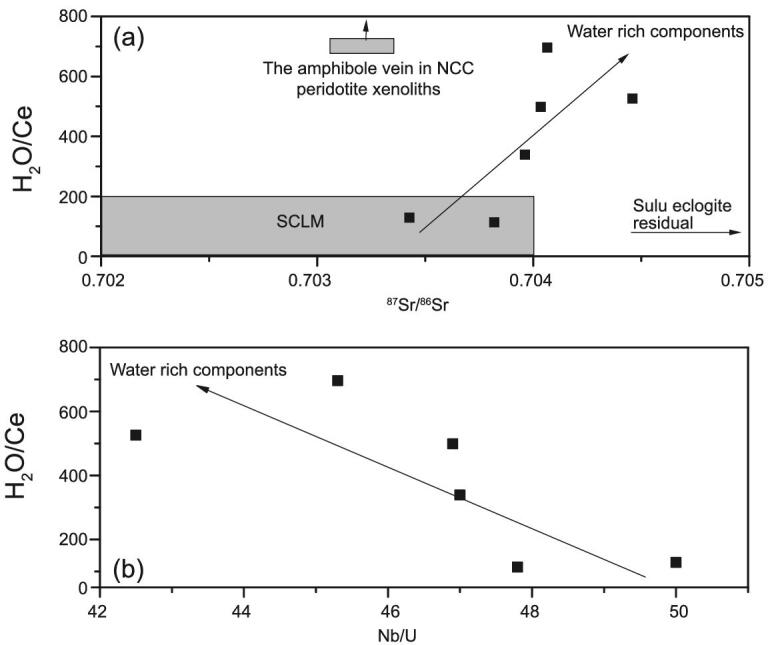
The correlation of H_2_O/Ce with the bulk-rock ^87^Sr/^86^Sr and Nb/U ratios of the Fuyanshan basalts in Shandong. Modified from Liu *et al*. [104].

The magma-water contents and corresponding H_2_O/Ce ratios provided new constraints on the origin of enriched components in the mantle sources of the Cenozoic eastern China basalts. As to the significance of the magma-water contents for identifying the source components, one typical example is the case of the Cenozoic alkali basalts in Shandong [104]. Both the recycled Pacific oceanic sediments and residual ancient lower continental crust (in the form of eclogite) that was produced by an earlier partial melting event have been suggested to be enriched components in the mantle source of the Cenozoic Shandong basalts [89,91,92,125]. Liu *et al*. [104] reported the water contents of the alkali basalts in one volcano (Fuyanshan volcano) in the same region. In addition to their exceptionally high water contents (see Regional distribution of H_2_O and H_2_O/Ce), the H_2_O/Ce ratios of these basalts were positively correlated with bulk-rock ^87^Sr/^86^Sr ratios and were negatively correlated with Nb/U ratios, respectively (Fig. 10) [104]. These trends demonstrate that the enriched components in the basalt sources should be enriched with water. Thus, the dry lower continental crust that experienced an earlier melting event before incorporation into the mantle sources could not be the appropriate candidate.

### The role of the Pacific subduction in the genesis of Cenozoic basalts in eastern China

Many trace elemental, radiogenic and stable isotopic evidences have been found to support the mantle sources of basalts from ∼110 Ma to the late Cenozoic in eastern China that contain components from subducted oceanic slabs [91,101,133,134]. This hypothetical oceanic slab has usually been assigned to the subducted Pacific slab, which was stagnant at the MTZ beneath eastern Asia [131,135]. Recently, the changes in basalt geochemistry (such as Eu/Eu*, ^87^Sr/^86^Sr and δ^18^O values) with eruption ages were determined [92,126,136–138] and they were suggested to reflect the secular contributions of different portions of the subducted Pacific oceanic crust in the mantle sources.

**Figure 11.**
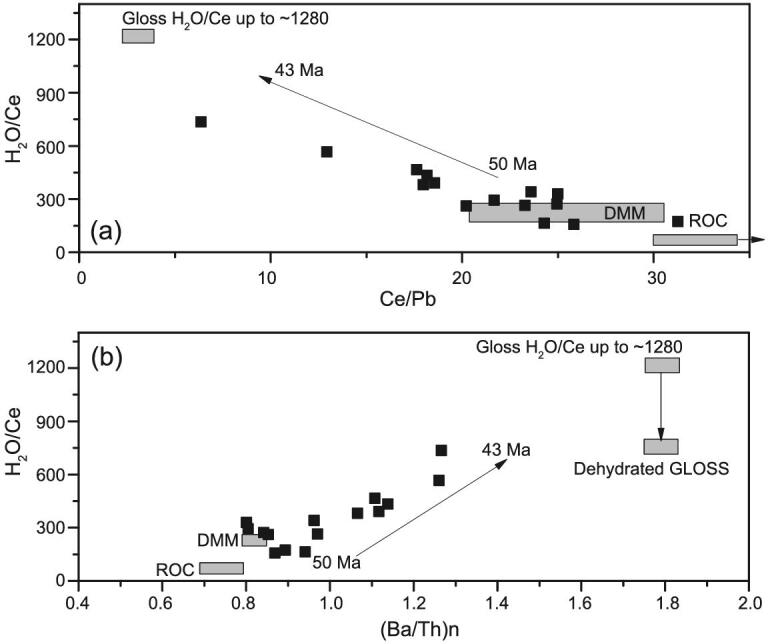
Correlations among H_2_O/Ce of the Cenozoic Shuangliao basalts and the bulk rock trace elemental ratios and radiogenic isotopic compositions. Gray squares represent the possible end members in the mantle source. Modified from Chen *et al*. [29].

Very recently, similar temporal variations in magma-water contents for several Cenozoic basalts in eastern China were resolved. Chen *et al*. [29] reported the water contents of the Shuangliao basalts in NNEC, which erupted from 50 to 43 Ma. As shown in Fig. 11, the younger basalts exhibit higher H_2_O/Ce ratios along with higher Ba/Th and lower Ce/Pb ratios, which reflect a secular contribution of the subducted oceanic sediments in the mantle sources. The similar temporal variations of H_2_O/Ce and other trace elemental ratios were also observed for the basalts in the Chaihe-Aershan (∼1.27–0.25 Ma) volcanoes of north-east China and the Zhejiang (26–17 and 11 Ma) volcanoes of SCB [103,105]. Overall, all those recycled oceanic components in the mantle sources of the Cenozoic basalts were dynamically incorporated, which would be best linked to the on-going subduction of the Pacific slab.

## WATER IN THE DABIE-SULU UHPM ECLOGITES: MEANS OF CARRYING CRUSTAL WATER INTO THE DEEP

### Water content and distribution in the Dabie-Sulu UHPM eclogites

It is well known that a great deal of aqueous fluid can be released from altered basalts and overlying sediments of the oceanic crust with increasing temperature and pressure during plate subduction. This process hydrates the overlying mantle wedge and usually generates syn-subduction arc magmatism on a large scale [139,140]. In contrast, aqueous fluids are less significant or absent during ultrahigh pressure metamorphism (UHPM) of the continental crust at mantle depths relative to that of the oceanic crust [141]. Because fluid is an important agent for element and isotope exchange, its presence or absence plays a critical role in the attainment and preservation of geochemical equilibrium between metamorphic minerals, and even the re-equilibration of geochronometric and geothermobarometric systems [142].

**Figure 12.**
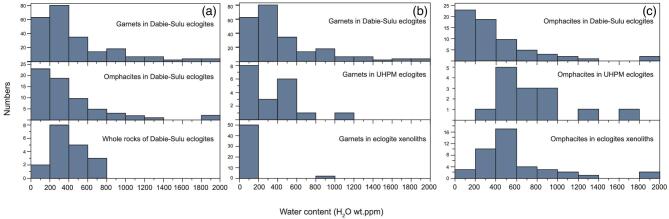
Water contents in the Dabie-Sulu eclogites and comparisons with other UHPM eclogites and eclogite xenoliths. (a) water contents in garnet, omphacite and bulk rocks from the Dabie-Sulu orogenic belt; (b) water contents in garnets from the Dabie-Sulu eclogites and other UHPM eclogites and eclogite xenoliths; (c) water contents in omphacites from the Dabie-Sulu eclogites and other UHPM eclogites and eclogite xenoliths. The dataset for the Dabie-Sulu eclogites is from Zhang *et al*. [143,144], Xia *et al*. [145], Sheng *et al*. [146], Chen *et al*. [147], Zhao *et al*., [148] and Zhao and Zhang [149]; and the dataset for other UHPM eclogites and eclogite xenoliths is from Aines and Rossman [150], Bell and Rossman [34], Langer *et al*. [151], Snyder *et al*. [152], Matsyuk *et al*. [153], Ragozin *et al*. [154], Bell *et al*. [155], Katayama *et al*. [156] and Skogby *et al*. [157].

The Dabie-Sulu orogenic belt in east-central China is the biggest exposed UHPM belt in the world, providing a natural laboratory to investigate the transportation and recycling of water. In principle, the major constituent minerals of UHPM eclogites are NAMs such as garnet, omphacite, rutile and silica phases. They are all stable phases in the P–T range typical of continental subduction-zone metamorphism and likely represent the primary water reservoirs in subducted slabs. FTIR analyses of NAMs in the Dabie-Sulu UHPM eclogites demonstrate that they contain a certain amount of water as structural hydroxyl locked in vacancies, which can be up to ∼1915 ppm for garnet, ∼1850 ppm for omphacite, ∼9590 ppm for rutile and ∼440 ppm for coesite [143–148]. The similar quantities of water in both garnet and omphacite indicate that they play equivalent roles in transporting surface water to mantle depths during continental subduction (Fig. 12a) [34,143–157]. Similarly to oceanic subduction, such transported water by subducted continents would cause prominent hydration (at least locally) in the mantle which they interact with.

Heterogeneous water contents in eclogite minerals were observed not only on the scale of the outcrop, but also among the different grains within the same hand-specimen. For instance, a >1000-ppm variation occurred for four to five garnet grains from ∼1-cm eclogite section [145]. Such small-scale heterogeneity clearly suggests very limited mobility of fluids during UHP metamorphism, and both subduction and exhumation processes of UHP rocks occurred over a short period of time [145]. Moreover, the decreased water content in the rims of some garnet and omphacite grains compared with the cores have been ascribed to hydroxyl exsolution upon the initial decompression exhumation of the UHPM eclogites. This process provides an efficient way to generate fluids during the early stage of exhumation and also promotes retrograde metamorphism [146]. The bulk-rock water contents are estimated to lie between 100 and 750 ppm [143–146]. In addition to structural OH, mineralogical studies by means of FTIR, transmission electron microscopy (TEM), backscattered electron (BSE) and laser Raman techniques indicate that NAMs contain significant amounts of water in the form of molecular H_2_O in fluid inclusions [145,158–161]. The fluid inclusions hosted by UHPM minerals may be either primary or secondary [162], thus providing an upper limit for the ability of transporting water into the mantle depth, while the structural hydroxyl in NAMs defines the lower limits.

### Comparison with upper-mantle eclogites

It is worth noting that either consistency or inconsistency of water contents has been observed in UHPM eclogites relative to mantle eclogite xenoliths hosted by kimberlites. For garnets, the water contents of UHP metamorphic rocks are significantly higher than in eclogite xenoliths (Fig. 12b). The prolonged residence in mantle conditions of eclogite xenoliths has the potential to result in a significant loss of water to the surrounding peridotitic mantle due to the usually much lower water contents in upper-mantle peridotites. Consequently, this can explain the lower water contents in garnets from eclogitic xenoliths [145]. In contrast, due to the presumably much shorter duration of tectonic exhumation, the UHPM eclogites may have a better capacity to preserve the water-rich character of their protoliths [145,146,163]. However, the water contents in omphacites from UHPM eclogites are comparable to those in eclogite xenoliths (Fig. 12c). This may reflect both the pressure and composition control on the incorporation of structural hydroxyl in crystal defects such as the Ca-Eskola components in M2-site vacancies [157,164]. Moreover, jadeite with water contents ranging from 100 to 1950 ppm and coherent variations in Na and Ca contents, as well as M2-site vacancies, have been reported for the Dabie UHPM jadeite-bearing quartzites [165]. These characteristics suggest that bulk mineral composition plays an important role in the incorporation of hydrogen [165]. Taken as a whole, the UHPM eclogites can contain higher water contents (>200 ppm) than MORB-sourced mantle peridotites that range from 50 to 200 ppm [166]. Therefore, the breakdown of such a water-rich subducted continental slab would have the potential to contribute to the ‘hidden' water-rich region in mantle depths as the OIB source.

Water in the form of structural hydroxyl is usually immobile in both hydrous and anhydrous minerals in UHPM conditions. The breakdown of the hydrous minerals in UHPM slices provides a dominant source for aqueous fluid during continental subduction-zone metamorphism [167,168]. Furthermore, numerous experimental studies have demonstrated that the solubility of structural hydroxyl in NAMs increases with pressure [156,164,169–175]. This, in turn, implies that the structural hydroxyl can be released from NAMs during exhumation. Therefore, significant amounts of aqueous fluids are available from the exsolution of structural hydroxyl and molecular water from NAMs in the initial stage of exhumation. The aqueous fluid then reacts with its host minerals resulting in their dissolution and recrystallization. Consequently, this metamorphic dehydration during subduction/exhumation would probably provide a sufficient amount of aqueous fluid for not only high-pressure eclogite-facies quartz veining, but also amphibolite-facies retrogression.

## SUMMARIZED CONCLUSIONS

- Although the main minerals in the lithospheric mantle of eastern China are nominally anhydrous, they usually contain a certain amount of water (up to several hundreds of ppm H_2_O) as hydroxyl groups in their structural defects. The temporal variation of the water content in the lithospheric mantle from the early Cretaceous (∼120 Ma) to Cenozoic (<40 Ma) appears tightly connected to the stability of the NCC (from its destruction to its consolidation), thus providing a clear case to show that the rheological changes induced by water in the lithospheric mantle affect the stability of continents.
- The heterogeneous water content in the Cenozoic lithospheric mantle beneath different blocks of eastern China was not only inherited from tectonic settings from which they came before having been amalgamated into a single continent, but was also affected by later geological processes that they experienced.
- The distinct water content between the lowermost crust and lithospheric mantle of eastern China and its induced rheological contrast at the base of the crust indicate that the continental crust–mantle boundary could behave either in a coupled or decoupled style beneath different areas and/or at different stages.
- The water contrast between the Precambrian and Phanerozoic lower crust of eastern China suggests a temporal evolution of the water content in the Earth's crust, different formation mechanisms of the continental lower crust or both. It is likely that the continental lower crust may have become gradually depleted with respect to its water content over geologic time, contributing to the stronger rheology and, therefore, preservation of the continental crust.
- The alkali basalts of eastern China demonstrate a heterogeneous distribution of the water content in the upper mantle and MTZ. Pacific plate subduction is likely the main process to introduce heterogeneities in the water content and other geochemical characteristics of the intra-plate magmas. Local and regional comparisons of the water content between the lithospheric mantle and basalt source demonstrate that the Cenozoic alkali basalts in eastern China were not sourced from the lithospheric mantle. Instead, the inferred high water contents in the mantle sources indicate that the Cenozoic eastern China basalts were likely sourced from the MTZ.
- The UHPM eclogites of the Dabie-Sulu orogeny can host up to thousands of ppm of water in nominally anhydrous garnets and omphacites, demonstrating that the ‘dry' continental crust may carry a certain amount of water back into the deep mantle by subduction. Such recycled continental crustal materials not only created a local water-rich zone, but also delivered continental geochemical signatures into the mantle, both accounting for the continental geochemical imprints in some OIBs and other intra-plate basalts.

## FUTURE ISSUES

- More data from other areas are necessary to examine certain conclusions drawn from the samples of eastern China, such as the higher water content in the lower crust than in the underlying lithospheric mantle and the more hydrous Precambrian lower crust than its Phanerozoic counterpart. In addition, the spatial and temporal distribution of the water content in the lithospheric mantle and its effect on continental evolution also require further exploration by collecting more data from other parts of the world.
- Pyroxenites are an important rock constituent in the upper mantle, and are likely the source lithology of some intra-plate basalts; in particular, local enrichment of Fe- and H-bearing pyroxenites may lead to regionally electrical anomalies, affecting the structure and some key properties of the upper mantle [176]. However, less attention has been paid to water in pyroxenites, and so far only Bizmis and Peslier [177] have reported water in six pyroxenite xenoliths with similar genesis (crystallized from basaltic magmas) from Hawaiian basalts. Due to the prominent role of water in the temperature, pressure and degree of melting and composition of the yielded melts, it is necessary to systematically study water in pyroxenites in different tectonic settings.
- Felsic rocks are the main constituents in UHPM orogens and the dominant assemblages in the shallower crust and the content and partitioning of water in minerals of felsic rocks should be investigated for a more comprehensive understanding of the recycling of water during the subduction and exhumation of continental plates and the storage of water in the crust and its exchange between different reservoirs.
- The magma-water contents provide new constraints on some important issues related to the production of intra-plate continental basalts, such as the nature of mantle sources (lithosphere vs. asthenosphere) and origin of enriched components. Available research relies on the established cpx phenocryst-based approach. The uncertainty of this method is relatively large and this method cannot be applied to the basalts free of cpx phenocrysts. Thus, in the future, a new methodology with better accuracy and wide applicability should be developed.
- As we demonstrated, the partition coefficient of water between opx and cpx in peridotites shows a range of values. In the future, these values should be more precisely explored with an explanation for scattering and whether there is any relationship with geochemical and physical variables.
